# Spatiotemporal dynamics of the proton motive force on single bacterial cells

**DOI:** 10.1126/sciadv.adl5849

**Published:** 2024-05-23

**Authors:** Anaïs Biquet-Bisquert, Baptiste Carrio, Nathan Meyer, Thales F. D. Fernandes, Manouk Abkarian, Farida Seduk, Axel Magalon, Ashley L. Nord, Francesco Pedaci

**Affiliations:** ^1^ Centre de Biologie Structurale, Université de Montpellier, CNRS, INSERM. Montpellier, France.; ^2^ Aix Marseille Université, CNRS, Laboratoire de Chimie Bactérienne (UMR7283), IMM, IM2B, 13402 Marseille, France.

## Abstract

Electrochemical gradients across biological membranes are vital for cellular bioenergetics. In bacteria, the proton motive force (PMF) drives essential processes like adenosine triphosphate production and motility. Traditionally viewed as temporally and spatially stable, recent research reveals a dynamic PMF behavior at both single-cell and community levels. Moreover, the observed lateral segregation of respiratory complexes could suggest a spatial heterogeneity of the PMF. Using a light-activated proton pump and detecting the activity of the bacterial flagellar motor, we perturb and probe the PMF of single cells. Spatially homogeneous PMF perturbations reveal millisecond-scale temporal dynamics and an asymmetrical capacitive response. Localized perturbations show a rapid lateral PMF homogenization, faster than proton diffusion, akin to the electrotonic potential spread observed in passive neurons, explained by cable theory. These observations imply a global coupling between PMF sources and consumers along the membrane, precluding sustained PMF spatial heterogeneity but allowing for rapid temporal changes.

## Teaser

Single-bacterium proton motive force shows rapid temporal and global spatial dynamics that are akin to passive neurons.

## INTRODUCTION

Evolved in all kingdoms of life, electrochemical gradients established out of equilibrium across bioenergetic membranes are a universal intermediate in energy conversion. Protons play a major role in processes such as cellular respiration and photosynthesis, leading to adenosine triphosphate (ATP) synthesis, wherein proton translocating enzymes generate a concentration gradient across specialized membranes that act as effective capacitors. The resulting stored electrochemical potential energy, the proton motive force (PMF), is composed of a difference in proton concentration and electrical potential across the membrane, as given by Mitchell’s classical chemiosmotic theory (*1*).

While long believed to be constant in time and space along the membrane, there is growing evidence pointing toward a rich spatiotemporal dynamical behavior of the PMF, although the biologically functional consequences remain poorly understood (*2*, *3*). The pioneering work performed in the 1980s by Skulachev and colleagues described long distance transmission of membrane voltage both in large multicellular populations of filamentous cyanobacteria and mitochondrial networks (*4*, *5*). In mitochondria, where the PMF regulates ATP generation, recent high-resolution studies have challenged this paradigm with evidences of local and dynamic PMF heterogeneity, arising from the complex geometry of the inner mitochondrial membrane, supramolecular organization, and microcompartmentation (*6*–*8*). In bacteria, where the PMF is known to regulate ATP synthesis (*9*), cell division (*10*), motility (*11*), antibiotic resistance (*12*), sporulation (*13*), and biofilm formation (*14*), recent observations also suggest a more complex spatiotemporal dynamical behavior of the membrane voltage and PMF. Rapid membrane depolarizations observed on single *Escherichia coli* cells (*15*), related to mechanosensing (*16*) and intercellular communication within biofilms (*14*), are indications of a temporally dynamic PMF. Moreover, it is well established that oxidative phosphorylation complexes dynamically organize into supramolecular micro-domains (*17*, *18*). Such spatiotemporal organization has been suggested to be beneficial for the stability of the individual complexes, to confine mobile electron carriers, and to limit the production of reactive oxygen species. Such vicinity of proton producers and consumers opens up the hypothesis of compartmentalization of the proton cycle, where a consequent local alteration of the proton concentration would result in a spatially heterogeneous PMF (*17*–*21*). As done in mitochondria, it is therefore necessary to revisit the classical view of homogeneous PMF in bacteria, with modern high-resolution techniques and at the single-cell level.

Despite its importance, the spatial and temporal dynamical behavior of the PMF in single bacteria remains poorly characterized. The challenge lies in bacteria’s micrometer-scale and their complex membrane composition, which precludes the use of classical techniques such as patch clamp, except at the expense of large perturbations to the cell envelope (*22*, *23*). Electric field–induced spectral shifts in carotenoids of photosynthetic bacteria have been used to infer the transient behavior of the transmembrane voltage at the population level (*24*, *25*). Techniques such as Nernstian and pH-sensitive dyes as well as genetically encoded pH and voltage sensors have recently enabled quantitative measurements at the single-cell level, pushing forward the nascent field of bacterial electrophysiology (*15*, *26*, *27*). While extremely valuable, these methods come with difficulties in transformation or labeling, as well as calibration of the often complex fluorophore response.

In this work, we turn to a biological probe to characterize the spatiotemporal PMF dynamics on single *E. coli* cells. The bacterial flagellar motor (BFM) is the PMF-driven nano-rotary motor located at the base of each flagellum, which drives cell motility in many motile bacteria (*26*, *28*, *29*). We use spatiotemporally structured laser excitation on single bacteria expressing the light-driven proton pump proteorhodopsin (PR) to controllably augment the PMF in space and time. Taking advantage of the linearity between BFM rotational speed and PMF (*30*–*32*) and building on previous works (*32*–*37*), we use the speed measurement of individual BFMs to resolve local PMF dynamics in the millisecond range on single cells.

Our measurements show that the capacitive response of the cell is asymmetrical and allows temporal PMF variations on the millisecond timescale, while spatially localized PMF perturbations are effectively homogenized over the entire cell. Such spread of the PMF occurs faster than diffusion can allow, in a process that links the electrical activity of bacteria with the electrotonic response of passive neurons. Such an electrotonic response, or subthreshold spread of voltage along the cell, independent from voltage-dependent elements and therefore distinct from the propagation of action potentials, is quantitatively explained by cable theory (*38*, *39*). This implies that a sudden increase in PMF due to a localized source is experienced synchronously (sub-millisecond) by all sinks on the membrane, prompting a global response to a local perturbation. Such global coupling between sources and sinks, as well as the consequent fast PMF homogenization, confirms the classical view of PMF and excludes the presence of a functional heterogeneity of the PMF resulting from membrane compartmentalization. The complex arrangement of the mitochondrial membrane, where PMF heterogeneity appears possible, marks the difference with respect to the geometrically simpler bacterial membrane.

## RESULTS

**Fig. 1.**
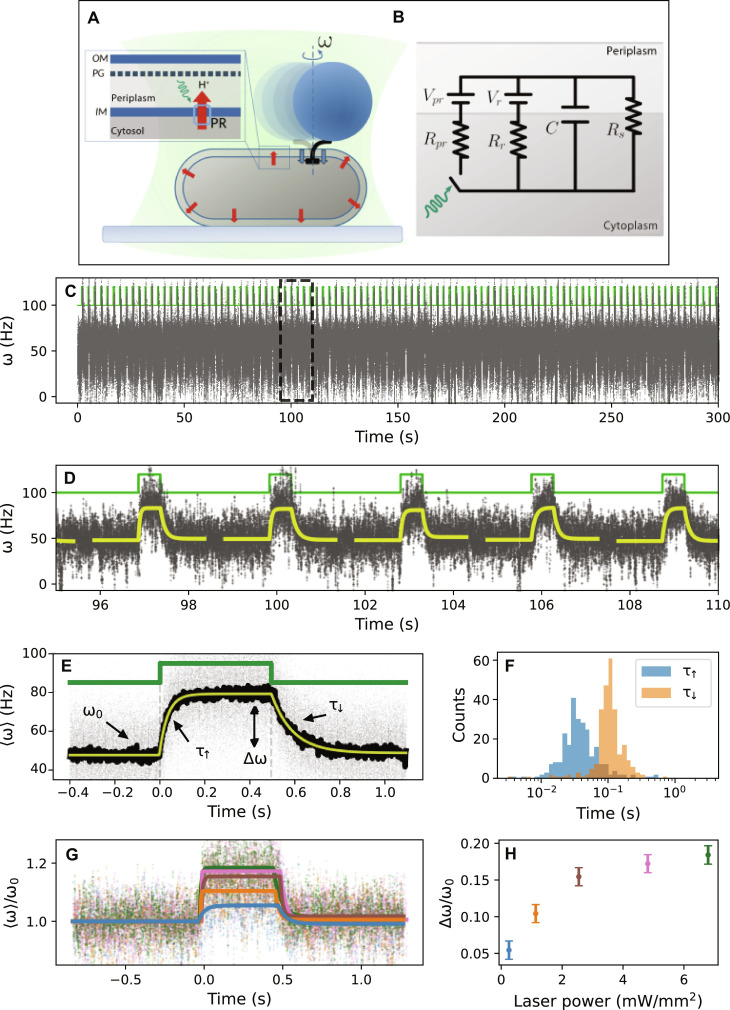
Temporal PMF dynamics on a single *E. coli* cell, probed by BFM rotation and PR excitation. (**A**) Schematic of the experimental assay. A cell is attached to the coverslip, and a 600-nm bead is bound to the truncated flagellar filament. Blue arrows, PMF consumption by the BFM; red arrows, PMF generated by the laser-activated PR. Inset: Schematic of the membrane (IM, inner membrane; PG, peptidoglycan; OM, outer membrane; PR, proteorhodopsin). (**B**) Cell circuit model. PR and respiratory complexes are modeled as voltage sources *V_pr_* and *V_r_* with internal resistances *R_pr_* and *R_r_*. *C*, membrane capacitance; *R_s_*, PMF sinks. PR excitation opens and closes the switch. (**C**) BFM speed (gray) induced by on-off rectangular laser pulses (green). (**D**) Zoom from a region in (C) indicated by dashed lines. Yellow, piecewise exponential (Eq. 6 and Materials and Methods) fit to each speed transition. (**E**) Overlay of 100 laser-synchronized transitions (gray), mean response (black), and fit of the mean (yellow). The motor speeds up upon a laser pulse from its steady-state speed ω_0_ to a excited steady-state speed ω_1_ = ω_0_ + Δω. τ_↑_ and τ_↓_, relaxation times. (**F**) Histogram of τ_↑_ and τ_↓_, extracted from the fits in (C). (**G**) Average (dots) and fit (lines) of dozens of normalized speed transitions on a single motor [on a different cell from (C) to (F)], where different colors show different laser powers. (**H**) Increase in speed as a function of laser power, extracted from (G).

Our measurements were based on a modification of the BFM bead assay, typically used to study the dynamics of the motor (*29*, *40*). We measured the BFM angular speed via bright-field microscopy [with up to a 20-kHz sampling rate; (*41*)] by tracking the recorded holograms of the off-axis rotation of 600-nm-diameter beads attached to flagellar stubs of individual immobilized *E. coli* cells (see Fig. 1A and Materials and Methods for details). We simultaneously manipulated the PMF of PR-expressing cells via laser excitation (see Materials and Methods and section S1 for details), producing a local excess of external proton concentration controlled by the spatiotemporal profile of the laser beam.

### PMF temporal dynamics

We first probed the temporal response of the system by monitoring BFM speed during periodic, spatially homogeneous laser illumination events. Under the assumption that PR is homogeneously distributed in the inner membrane (see section S9), laser illumination creates a homogeneous excess of external proton concentration, globally increasing the PMF of the cell under study. At each excitation pulse, we observed the motor accelerating from the native steady-state speed ω_0_ to a higher speed ω_1_, due to the PR-generated PMF increase (Fig. 1, C to E). We note that the number of the active stator units in the motor is dynamic, mechanosensitive, and PMF dependent (*29*, *34*, *42*, *43*). By tuning the laser pulse duration and the time between pulses, we aimed to avoid an increase in the time-averaged PMF and thus avoid an increase in stator number. We recorded up to thousands of transitions from ω_0_ to ω_1_ on single motors (see Fig. 1C), where 〈ω_0_〉 (and thus stator number) remained constant.

By overlaying and averaging hundreds to thousands of laser-synchronized speed transitions, we obtained the average PR-induced speed response with high signal-to-noise ratio (Fig. 1E, black line), which strongly evokes the charge and discharge of a capacitor. Following previous works (*33*, *35*, *37*, *44*), we thus modeled the cell by a single lumped electric circuit (Fig. 1B), where the inner membrane acts as a capacitor separating the cytoplasm and periplasm [we assume the periplasm and extracellular volume to be in thermodynamic equilibrium due to the high permeability of the outer membrane; (*45*)]. Analogous to a voltmeter, the BFM produces a speed proportional to the voltage across the capacitor. Proton sinks in the membrane (e.g., ATP synthase) act as resistances in parallel and are combined in the resistance *R_s_*. Native membrane-embedded respiration complexes, which act as proton sources, are modeled as a single voltage source *V_r_* with internal resistance *R_r_*. Similarly, once excited, PR transporters act as a light-controlled proton source, modeled by a light-triggered switch in the circuit with a voltage source *V_pr_* and internal resistance *R_pr_*. The analytical expression for the transmembrane voltage, established across the capacitor and detected by the BFM (solved from the circuit in Fig. 1B), can be written by separating the charging *V*_↑_(*t*) and discharging *V*_↓_(*t*) processes, upon inclusion of the PR arm under laser excitation, as

$$V_{↑}\left(t\right)=V_{1}-\left(V_{1}-V_{0}\right)e^{-t/\tau_{↑}}$$ (1)

$$V_{↓}\left(t\right)=V_{0}-\left(V_{0}-V_{1}\right)e^{-t/\tau_{↓}}$$ (2)

where $V_{0}=R_{↓}\frac{V_{r}}{R_{r}}$ and $V_{1}=R_{↑}\left(\frac{V_{r}}{R_{r}}+\frac{V_{\mathrm{pr}}}{R_{\mathrm{pr}}}\right)$ are the native and excited steady-state voltages, respectively, with equivalent resistances $R_{↑}={\left(\frac{1}{R_{r}}+\frac{1}{R_{s}}+\frac{1}{R_{\mathrm{pr}}}\right)}^{-1}$ and $R_{↓}={\left(\frac{1}{R_{r}}+\frac{1}{R_{s}}\right)}^{-1}$ , and where the characteristic relaxation times can be written as τ_↑_ = *R*_↑_*C* and τ_↓_ = *R*_↓_*C*, for the charging (↑) and discharging process (↓), respectively. We note that the solution predicts two distinct relaxation times for the charge and discharge and that τ_↓_ > τ_↑_. This can be understood by the fact that the arm of the circuit associated to PR, present only during the charging due to laser illumination, brings the extra resistance *R_pr_*, which makes the equivalent resistance *R*_↑_ < *R*_↓_.

The experimental mean response of the motor to the pulsed PMF excess was fit by a continuous piecewise function built with *V*_↑_(*t*) and *V*_↓_(*t*) as shown by the red line in Fig. 1E (each synchronized with their relative laser pulse edges, such that, at the laser-off time, the two functions are equal; see Materials and Methods). In agreement with the prediction from the electric circuit model, we find that τ_↓_ > τ_↑_, as shown by a fit of the mean transition in Fig. 1E (black line; τ_↓_ = 92 ± 0.5 ms and τ_↑_ = 36 ± 0.5 ms; see section S3 for uncertainty estimation). This feature was robustly observed in all the measured motors (total of 12 motors; see global statistics in section S4) and is in direct agreement with the prediction of the electric circuit model. Fits of each individual transition, although noisier, also show this trend (Fig. 1F).

In Fig. 1 (G and H), we show that the speed change Δω = ω_1_ − ω_0_ increases with laser power, up until a saturation, as seen previously (*33*). This could either be explained by the fact that *R_pr_* decreases to a finite value with light intensity, reflecting the limiting rate of PR pumping (*33*, *35*), or by an intrinsic saturation in the motor. Analogously, oxygen depletion, which limits proton pumping of respiratory complexes (see Materials and Methods), can be modeled by an increasing *R_r_* (see section S10). The model shows that, in this case, the difference *V*_1_ − *V*_0_, proportional to Δω, increases in line with our observations. The motor response to the periodic PR excitation (Δω) was observed to increase, while ω_0_ decreased, with the time spent by the cells in the sealed volume of the flow slide (see Materials and Methods). This is in line with previous observations (*33*, *35*) and may be related to a decreased activity of respiratory complexes with a decrease in available oxygen. In support of this, we also observed an increase in Δω in the presence of low concentrations of NaN_3_, a bacterial respiration inhibitor (see section S10). It has also previously been shown that PR-induced photocurrent increases as the absolute value of the transmembrane voltage decreases (*46*).

### PMF spatial dynamics

**Fig. 2.**
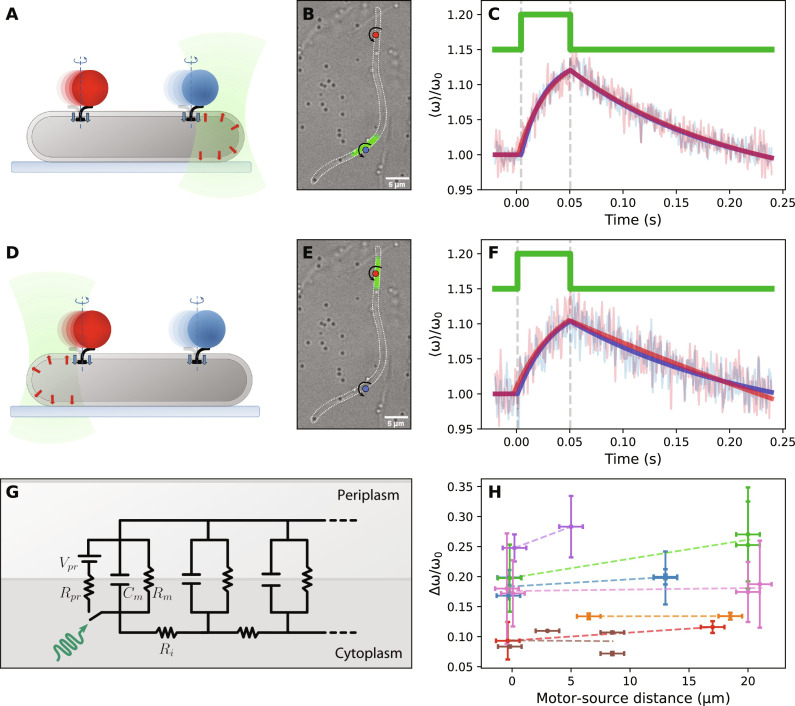
Spatial PMF dynamics. (**A**) Local illumination of a filamentous *E. coli* cell, where activating PR triggers a local PMF increase. The speed response of two motors located close (blue) and far (red) from the source was measured. (**B**) Microscope image of the assay depicted in (A), where the green spot indicates the portion of the cell illuminated by the laser and colored circles indicate the rotating beads attached to truncated filaments (**C**) Average of 1493 (red) and 1793 (blue) laser-synchronized speed transitions (faint lines) and the fits (dark lines) of the two motors shown in (B) located near (blue) and far (red) from the laser (green). (**D** to **F**) Same as (A) to (C) with the local illumination at the opposite motor. (F) Average of 751 (blue) and 690 (red) transitions. (**G**) *E. coli* circuit model based on cable theory and consisting of sub-circuits in series, each modeling a small section of the cell. A non-illuminated module is represented by the membrane capacitance (*C_m_*) and sinks (*R_m_* = *R_s_*) whereas the illuminated module includes the PR arm (with voltage source *V_pr_*, resistance *R_pr_*, and light-activated switch) as for the circuit in Fig. 1A. *R_i_* is the cytosolic resistance, while the extracellular medium is considered equipotential. The transmembrane voltage becomes now a function of both space and time. (**H**) Relative increase in speed as a function of the distance between the source and the motor, where measurements on motors of the same cell are indicated by points of the same color, for which a linear fit (dotted line) is shown to guide the eye.

We then asked whether a spatial heterogeneity of the PMF can exist and be detected by our measurements. To maximize the possible effect of an inhomogeneous PMF, we used filamentous and PR-expressing *E. coli* cells with lengths up to 30 μm (see Materials and Methods for details), which we periodically excited at only one pole, using a 8-μm-diameter laser spot (see section S2). We chose filamentous cells where two functional motors were labeled by beads, giving two PMF reporters at different distances from the proton source (up to a maximum of 20 μm), which was spatially and temporally defined by the intersection of the laser beam with the cell (Fig. 2, A and B). As a control, we recorded the two motor speeds during localized illumination of each cell pole, such that each motor played the role of both the distal and proximal sink. The measurements (Fig. 2, A to F) show that both motors display the same charging-discharging behavior described above, with a temporal profile that is notably similar, within error, independent of their distance from the source. Two features, in particular, can be highlighted: (i) the response of both motors starts at the laser-on edge with no resolved delay (within our time resolution of 2 ms; see section S3); and (ii) once normalized to the steady-state value ω_0_, the speed increase is the same for both motors (Fig. 2, C and F). Both features were observed in all measurements at constant laser power (seven filamentous cells), as summarized in Fig. 2H, and at increasing laser powers (see section S5). We conclude that an excess of PMF generated locally can propagate for tens of micrometers in less than a few milliseconds with no measurable loss, such that all sinks within these distances are affected identically.

**Fig. 3.**
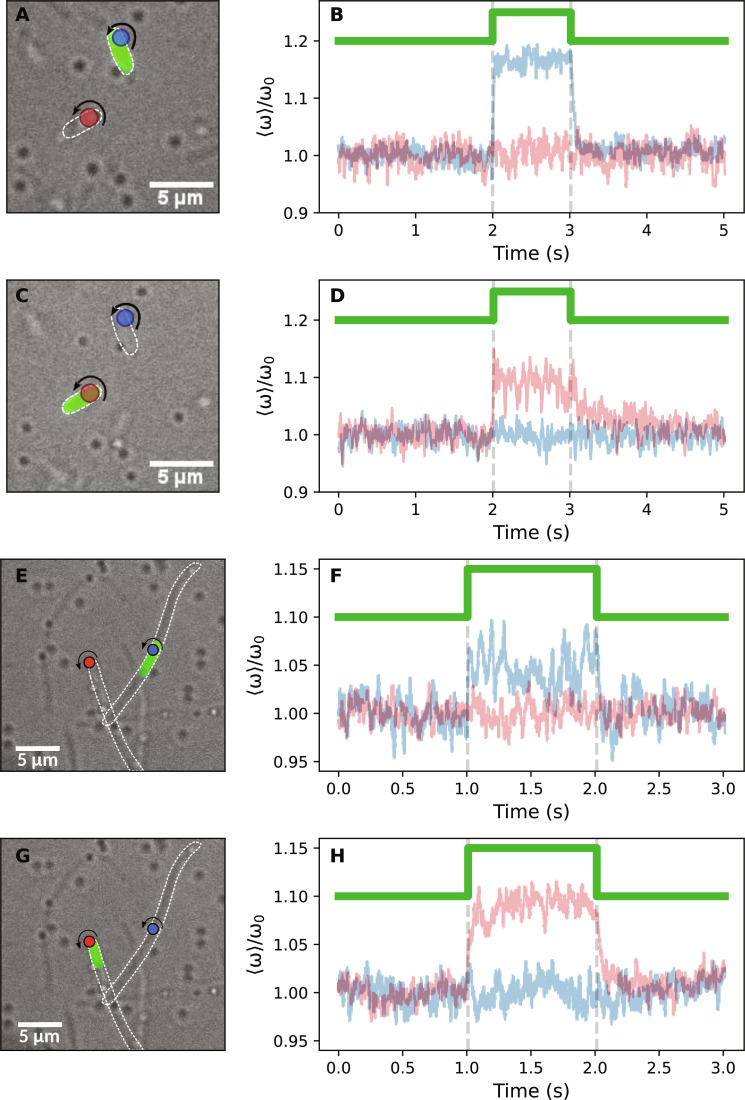
Effect of PR excitation on neighboring and overlapping cells. (**A**) Two bacteria, each with a labeled motor, are separated by ∼3 μm; the cell labeled by the blue bead is illuminated with the edge of the laser spot (depicted in green). (**B**) Normalized average speed response of hundreds of transitions of the two motors, labeled by the same colors. (**C** and **D**) Same as (A) and (B), but the laser excites the cell labeled by the red bead. (**E** and **F**) Image and speed traces for two filamentous overlapping cells, each with a labeled motor. The cell labeled by the blue bead is illuminated by the edge of the laser. (**G** and **H**) The same as (E) and (F), but the cell labeled by the red bead is illuminated. The colors of the traces correspond to the color of the bead.

We then wondered whether PR, pumping protons from one cell, could locally and transiently acidify the solution around cells in close proximity, affecting their PMF, akin to the long range electric effect of other ions (*14*). We selected pairs of nonfilamentous cells located less than 8 μm from each other, both with a bead-labeled functioning motor. Periodically illuminating only one cell at a time, we observed that only the motor belonging to the illuminated cell increased its speed following the laser (Fig. 3, A to D). Similarly, we sequentially illuminated motors on two overlapping filamentous cells, and we observed that only the motor of the illuminated cell showed PR-induced acceleration (Fig. 3, E to H), irrespective of the laser position along the cell. These observations indicate that the PMF excess produced across one membrane cannot power a motor on a separate cell, even if the two cells are in close proximity or in contact.

## DISCUSSION

In the first part of this study, we probed the temporal response of the system via homogeneous PR excitation, showing that PR can perturb the PMF and that the BFM can report upon PMF dynamics, both at the millisecond timescale. We observed relaxation times (τ_↑_ and τ_↓_) on the order of tens of milliseconds, one to three orders of magnitude lower than previous measurements using the BFM (*33*, *35*, *36*). As it is now known that the number of bound and active stators is proportional to the PMF (*26*), it is likely that the previously measured timescales were dominated by dynamic stator exchange. By carefully avoiding the effect of stator dynamics, the shorter timescale that we resolve reports upon the cellular electrical response. This timescale is unaffected by proton pumping by PR (with the laser off, τ_↓_ is not influenced by PR), nor is it severely limited by the elasticity of the flagellar hook (see Materials and Methods). The electric circuit model of Fig. 1B predicts an asymmetry in the two characteristic times of the system, which we systematically observe in our experiments (i.e., τ_↓_ ≃ 100 ms and τ_↑_ ≃ 30 ms; see Fig. 1F). By using the measured values of τ_↑_, τ_↓_, ω_0_, and ω_1_, together with an estimate of the membrane capacitance (*C* ≃ 10^−14^F) and of the respiration source (*V_r_* ≃ 360 mV) (*33*, *47*), we use the analytical results obtained from the theoretical solution of the circuit to determine the values of all the cellular electrical parameters (see section S6): *V_pr_* ≃ 62 mV, *R_pr_* ≃ 10^12^ ohms, *V_r_* ≃ 360 mV (*33*), *R_r_* ≃ 9 × 10^13^ ohms, *R_s_* ≃ 10^13^ ohms, *C* ≃ 10^−14^F (*33*, *47*), *V*_0_ ≃ 37 mV, and *V*_1_ ≃ 60 mV.

In the second part of this work, by controlling the spatial position of the PMF source, we observed that the response of the BFM is independent of its distance from the proton source, up to ∼20 μm. It is worth noting that, among ions, protons behave peculiarly (*48*), and the classical model of a “delocalized” PMF (where protons immediately equilibrate into the bulk solution) has been challenged by numerous studies of proton dynamics along membranes. Despite variations in quantitative results and ongoing discussions about the underlying mechanisms, many observations indicate that protons translocated across membranes can remain confined and in close proximity to the membrane (*8*, *17*, *49*–*57*). Efficient proton diffusion can then occur in two dimensions with a high diffusion coefficient, comparable to bulk [where protons have the highest diffusion coefficient among ions, *D_H_* = 9 × 10^−5^ cm^2^/s, due to the Grotthuss mechanism; (*58*)]. While such a “localized” model of PMF provides a coherent picture of energy transduction, evidence comes mainly from controlled in vitro systems; measurements in vivo, and particularly in bacteria, are lacking. Proton diffusion measurements on synthetic lipid bilayers show a substantial deviation from classical diffusion at short timescales (<100 ms) (*54*, *59*). Our measurements could be seen as in line with this “anomaly” at the millisecond scale in living bacteria. As we observe that the transfer of PMF does not occur across neighboring or intersecting cells, our results could be explained by a surface-localized PMF transmission, although the need of a more direct proof remains.

Note that, despite their different physical nature, proton concentration ρ(*x*, *t*) and transmembrane voltage *V*(*x*, *t*) (functions of space *x* and time *t*), both contributing to the PMF, obey the same diffusion equation, which can be generally written in one dimension as

$$\tau_{A}\frac{\partial A}{\partial t}=\lambda_{A}^{2}\frac{\partial^{2}A}{\partial x^{2}}-A+F_{A}$$ (3)

where *A* = ρ(*x*, *t*) or *V*(*x*, *t*); τ*_A_* and λ*_A_* are the characteristic time and length, respectively; and *F_A_*(*x*, *t*) is the source term. Taking *A* = ρ, Eq. 3 describes concentration diffusion in the presence of losses from sinks (with first order rate *k_s_*) and gains from sources (with rate *k_pr_*), where the parameters can be expressed as τ_ρ_ = 1/*k_s_*, $\lambda_{\rho }=\sqrt{D_{H}/k_{s}}$ , and *F*_ρ_ = *k_pr_*/*k_s_* (*59*). On the other hand, taking *A* = *V*, Eq. 3 successfully models the transmembrane voltage in the different context of cable theory, developed for the electrophysiology of unmyelinated passive neuronal axons (with voltage below the action potential threshold) (*38*, *39*, *60*). Here, the cylindrical cell is described by an equivalent electric circuit made by serially stacked resistive-capacitive (RC) sub-circuits, each modeling a small section of the cell (Fig. 2G). Solving the circuit, the theory provides the deviation from steady state of the transmembrane voltage *V* (*38*). The parameters are here related to the electric components of the circuit as τ*_V_* = *R_m_C_m_* and $\lambda_{V}=\sqrt{R_{m}d/\left(4R_{i}\right)}$ , where *R_m_* (units of ohm·m^2^) is the membrane resistance across a unit area, *R_i_* (ohm·m) is the intracellular volume resistivity, *C_m_* (F/m^2^) if the capacitance per unit area, *d* the cell diameter, and *F_V_*(*x*, *t*) is the source, resulting from an injected current (which, on axons, can be localized by the use of micropipettes). Providing the deviation from steady state, the circuit model (Fig. 2G) does not include a branch for respiration, which simply offsets the solution.

**Fig. 4.**
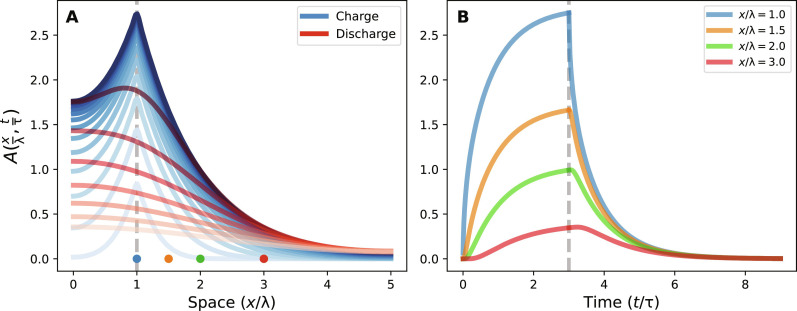
Analytical solution of the cable equation. The solution of Eq. 3 is shown as a function of the normalized variables (*x*/λ and *t*/τ). The source is a delta function centered in *x*/λ = 1, active between *t* = 0 and *t* = 3τ, and the cell has a length of 5λ. (**A**) Behavior of the solution in space, where light to dark blue lines indicate different times during charging (*t* = [0,3τ]), and dark to light red lines indicate different times during discharging (*t* = [3τ,6τ]). (**B**) Temporal profiles of *A*(*x*, *t*) at positions *x*/λ indicated by the colored dots in (A).

An analytical solution of Eq. 3 for a spatiotemporally localized source can be found both in the context of diffusion (heat transfer) (*61*) and in cable theory (see sections S7 and S8) (*39*). As shown in Fig. 4, during the charging and as a function of space, the solution peaks at the source location and exponentially decays with characteristic length λ (dropping the index for simplicity; see Fig. 4A). During the discharge, the peak is smoothed before the solution reaches zero everywhere at long times. During charging and as a function of time, the solution saturates to a plateau everywhere within a few characteristic times τ (Fig. 4B). As the distance from the source increases, both a delay in the rising edge and a decrease in the plateau occur. During discharge, the solution shows an exponential decay, with a delay in the falling edge that increases with distance to the source. We note that the solution of classical cable theory displays symmetric characteristic times during charge and discharge (τ_↑_ = τ_↓_) due to the fact that the source *F*(*x*, *t*) is modeled as a current source. We find that by instead modeling the source as a battery *V_pr_* with internal resistance *R_pr_* (Fig. 2G) or by introducing a saturation mechanism in the pumping rate [*k_pr_* = *k_pr_*(ρ)] in two-dimensional diffusion simulations, the observed asymmetry (τ_↓_ > τ_↑_) is reproduced (see section S8). This general solution is instrumental to discriminate the relative roles of concentration and voltage in our measurements, as discussed below.

Concentration diffusion alone cannot explain our data. If we assume that the measured characteristic timescale τ*_m_* = 〈(τ_↑_, τ_↓_)〉 = (30 to 100) ms is due to concentration diffusion (i.e., τ*_m_* = τ_ρ_ = 1/*k_s_*) and considering the largest diffusion coefficient for protons in bulk (*D_H_*), then we estimate the diffusion characteristic length as

$$\lambda_{\rho }=\sqrt{D_{H}/k_{s}}≃\left(15\;\text{to}\;30\right)\mathrm{\mu m}$$ (4)

We note that, in the presence of mobile buffer molecules, the effective diffusion of interfacial protons is further reduced. In vitro, millimolar buffer concentrations have been shown to remove surface protons, slowing their diffusion (*52*, *54*). Not knowing the concentration nor the effect of the buffer on proton diffusion in the bacterial periplasm, we consider the fastest diffusion via *D_H_*. However, this would set the maximum distance between two experimentally probed motors (∼20 μm) at about one characteristic length λ_ρ_. First, at such a distance, as shown from the analytical solution (comparing the blue and green curves of Fig. 4), we expect a delay in the response of the distal motor on the order of ∼0.1 τ_ρ_, i.e., (3 to 10) ms, which we do not observe within our millisecond experimental time resolution (see section S3). More evidently, we expect a clearly resolvable difference between the plateaus reached during charging between the motor located at the source and the distal motor (30 to 50%; see the blue, orange, and green lines in Fig. 4B). We do not observe this difference, and the two motors behave identically. We thus conclude that proton diffusion alone is not sufficient to explain the observed rapid and lossless transmission of PMF. To explain the observations, the diffusion coefficient would have to be >10*D_H_*, which is nonphysical.

Instead, if we hypothesize that the measured characteristic timescale τ*_m_* is due to voltage dynamics (i.e., τ*_m_* = τ*_V_* = *R_m_C_m_*), from the values *C_m_* ≃ 1 μF/cm^2^ (*33*, *47*), *R_i_* ≃ 100 ohm·cm (*38*, *47*), and *d* = 1 μm, then we can estimate *R_m_* ≃ (1 − 10) ohm·m^2^ and the voltage characteristic length

$$\lambda_{V}=\sqrt{\frac{R_{m}}{R_{i}}\frac{d}{4}}≃\left(0.5\;\text{to}\;1.5\right)\text{mm}$$ (5)

In this scenario and compatible with our data, the experimental maximum distance between a motor and the PR-induced source (∼20 μm) is a small fraction of λ*_V_*. Therefore, as the source creates a local proton excess, the transmembrane voltage *V*(*x*, *t*) takes a few tens of milliseconds (τ_↑_) to reach the new steady state, which, although exponentially decaying in space, is nearly constant over the length of the cell, evolving in time identically everywhere along the cell. Millimeter-scale characteristic lengths of the transmembrane voltage are also found in passive neuronal axons (*38*, *39*, *60*, *62*) and confirm the pioneering observations of long distance PMF transmission in millimeter-scale multicellular filamentous cyanobacteria by Skulachev and colleagues (*5*). The possibility for cells and tissues to develop currents through and around themselves, via asymmetrical distributions of sources and sinks, has also been recognized (*63*). At the microscale, beyond the classical electric circuit description of cable theory, we note that the fact that proton diffusion is not fast enough to explain our observations calls for other mechanisms leading to enhanced proton transport. A complete theoretical understanding of the interplay between charge distribution and transmembrane voltage in the small volume of a bacterium will likely benefit from an electro-diffusion treatment via the Poisson-Nernst-Planck equations, as recognized for mitochondria and submicron neuronal compartments (*64*, *65*).

In conclusion, we have found that the bacterial PMF can exhibit temporal dynamics with characteristic times in the tens of milliseconds, depending on the conductance of the ion channels involved. Spatially, our measurements imply that sources and sinks are globally coupled, effectively experiencing the same value of PMF, within less than milliseconds, independently of their location in the membrane. This can be explained by the millimeter-scale characteristic length of the transmembrane electrotonic voltage spread from a source over the micron-scale bacterial cell. A spatially homogeneous PMF implies that sinks do not compete for protons and that their consumption rate is independent of their position in the membrane, refuting the hypothesis of local proton circuits in bacteria, and in line with the classical view of spatially homogeneous PMF. For peritrichious bacteria, this feature may be crucial, as theory suggests that to form a coordinated flagellar bundle, each of the multiple membrane-bound motors must have nearly equal rotation rates (*66*). The rapid homogenization of the PMF suggests that the observed clustering of respiration complexes observed in bacteria (*17*, *18*, *20*) is not associated with a localized enhancement of PMF. Instead, clustering may contribute to an enhanced efficiency of electron transport between complexes, subsequently facilitating proton pumping.

With similar characteristic length and timescales, despite their vast difference in size, the electrophysiological properties of bacteria and neurons are intriguingly similar.

## MATERIALS AND METHODS

### Bacteria strain and culture

We used *E. coli* strain MT03, a derivative of RP437 where *cheY* is deleted, producing counterclockwise only BFM rotation, and *fliC* is replaced with a mutant *fliC^st^*, producing “sticky” filaments and enabling the attachment of polystyrene beads. This strain was transformed with the *pBAD24-PR* plasmid (amp^R^, arabinose induced) containing SAR86 δ-PR. Cells were inoculated from a single colony on a plate into LB (Bacto-Tryptone, 10 g/liter; yeast extract, 5 g/liter; and NaCl, 10 g/liter) containing ampicillin (100 μg/ml) and grown aerobically overnight at 35°C, shaking at 200 rpm. The overnight culture was diluted to optical density at 600 nm (OD_600_) of 0.05 with LB, PR expression was induced with 0.01% arabinose, and 10 μM ethanolic all-trans-retinal was added as the necessary cofactor. Cells were grown at 35°C and harvested during mid-log growth phase, at an OD_600_ of 0.6 to 0.8. Filamentous cells were prepared by adding cephalexin (60 μg/ml) at early-log phase and grown for another 3 hours.

### Sample preparation

Flagella were mechanically sheared by passing 1 ml of cell culture back and forth between two syringes connected by two 21-gauge needles and a thin tube (*31*). Cells were then centrifuged at 3000 rpm for 2 min. After discarding the supernatant, the cells were washed and resuspended in motility buffer [MB; 10 mM potassium phosphate, 0.1 mM EDTA, and 10 mM lactic acid (pH 7.0)]. Custom-made tunnel slides were made of two coverslips separated by a piece of parafilm with a “tunnel” cut from its interior. We flushed 100 μl of poly-l-lysine (Sigma-Aldrich, P4707) into the tunnel slide, incubated for 2 min, and then flushed through 200 μl of MB. The cell suspension (200 μl) was then flushed through the tunnel slide, and cells were allowed to settle on the coverslip for 10 min. Unattached cells were washed out with MB. Last, 600-nm polysterene beads (Sigma-Aldrich, LB6; 300 μl of a 1/300 dilution into MB) were flushed through. Beads were allowed to spontaneously bind to the sticky truncated filaments, and, after about 10 min, unattached beads were washed out with MB. The consensus view of the relationship between motor speed and PMF is that of linear proportionality with zero intercept (*31*). Such linear relationship has been shown to hold both for large loads (beads of 1-μm diameter), as well as for smaller loads (400-nm bead). As we have found that our 600-nm beads provide a good signal to noise ratio upon PR excitation and not expecting marked differences, we have not run a systematic study of bead size. To improve the signal-to-noise of the PR excitation induced transitions, we worked in conditions where oxygen was partially depleted: Cells were left in the sealed tunnel slide for about 1 to 2 hours (depending on the bacterial concentration in the sample) before starting the acquisitions. Experiments were performed in MB at 22°C.

### Microscopy

The custom-built microscope used for all experiments is shown in section S1 and described in (*41*). The sample was continuously illuminated with a 600-nm light-emitting diode (Thorlabs, M660L3) and imaged with a 100×, 1.45 numerical aperture, objective (Nikon) onto a complementary metal-oxide semiconductor (CMOS) camera (Optronics CL600x2/M) at 5 to 20 kHz, with a pixel size of 88 nm. PR was excited by a 552-nm laser focused onto the back focal plane of the objective after being spatially limited by an iris placed on a conjugated plane. The laser diameter at the sample plane, measured by the fluorescence of a thin layer of Rose Bengal (Sigma-Aldrich, 198250-5G), was ∼8 μm (see section S2 for details), and the intensity at the exit of the objective was 108 mW/mm^2^ for PMF temporal dynamics experiments and 6 mW/mm^2^ for PMF spatial dynamics experiments. Periodic PR excitation was achieved via an acousto-optic tunable filter (AOTFnC-400.650-TN, AA Opto-electronic) to produce a periodic train of on-off rectangular laser pulses. In the temporal dynamics experiments, the cycle lasted 2.5 s with the laser on for 0.5 s, whereas, in the spatial dynamics experiments, the cycle lasted 250 ms with the laser on for 50 ms. The position of the laser with respect to the bacteria and rotating beads was determined before each measurement by imaging the sample onto an electron-multiplying charge coupled device (iXon Ultra 897, Andor, 109 nm per pixel) via a removable mirror and using the autofluorescence of the cell as a proxy for the 552-nm illumination. A small percentage of the modulated PR excitation laser was picked out and imaged onto the corner of the CMOS camera via an optical fiber to automatically synchronize the sampling of laser intensity and motor speed. We note that the 660-nm illumination light used for bead tracking is within the tail of the PR excitation spectrum, and it increases the BFM speed by ∼4%. As it remains illuminated for the entirety of our measurements, its effect is negligible.

### Data analysis

All data analysis was performed using custom LabVIEW and Python scripts. The *x*(*t*), *y*(*t*) positions of the rotating bead were determined by using a cross-correlation analysis of the bead image with a numerically generated kernel pattern (*67*). The drift of the circular trajectory was corrected by subtracting a spline interpolation of *x* and *y* from their respective raw values. The elliptical trajectories of the beads, assumed to be the projection of a tilted circle, were transformed into circles by stretching the minor axis of the ellipse. The bead angle was calculated as θ = tan^−1^(*y*/*x*), and the angular velocity as ω = *d*θ/*dt*. The speed trace was low-pass–filtered by the Savitzky-Golay algorithm (fifth order, 2- to 8-ms window). Motor speed transitions around a laser pulse were fit using a differential evolution fit algorithm with the following piecewise function

$$\omega (t)=\{\begin{array}{ll} \omega_{0} & \text{if}\;t\leq t_{0}\\ \omega_{0}+(\omega_{1}-\omega_{0})[1-e^{-(t-t_{0})/\tau_{↑}}]=u(t) & \text{if}\;t_{0}<t\leq t_{1}\\ \omega_{2}+[u(t_{1})-\omega_{2}]e^{-(t-t_{1})/\tau_{↓}} & \text{if}\;t_{1}<t \end{array}$$ (6)

where *t*_0_ (*t*_1_) is the time at which the laser is switched on (off); τ_↑_ and τ_↓_ are the characteristic times; and ω_0_, ω_1_, and ω_2_ are the steady-state speeds before switching on the laser, after switching on, and after switching off, respectively. The free parameters of the fit were (τ_↑_, τ_↓_, *t*_0_, *t*_1_, ω_0_, ω_1_, and ω_2_). Speed traces that showed obvious stator incorporation or dissociation events were manually excluded; while the case of variable stator stoichiometry is interesting, it is left for future studies. The distance between the region of the cell illuminated by the laser and the motor was measured using the laser excited autofluorescence of the cell and the center of the bead trajectory (see section S2 for details). We note that BFM bead assay measurements are intrinsically low-pass–filtered by the flagellar hook, the 60-nm extracellular polymer located at the base of the flagellum, which acts as a torsional spring between the motor and the observed load. Given its torsional stiffness (*68*), the corresponding relaxation time for a 600-nm bead tethered to the hook is about 2 ms, which is smaller than the measured characteristic times (τ_↑_ and τ_↓_), and therefore should not severely affect the results.

## Supplementary Materials

### This PDF file includes:

Sections S1 to S10 Figs. S1 to S8
