# Supplementary material for: Spatiotemporal dynamics of the proton motive force on single bacterial cells
Source: Sci Adv. 2024 May 23;10(21):eadl5849. doi: 10.1126/sciadv.adl5849 (PMC11114223; doi:10.1126/sciadv.adl5849)
Supplement: Supplementary file 1 — Sections S1 to S10 Figs. S1 to S8 [file sciadv.adl5849_sm.pdf]

Science Advances
AAAS

Supplementary Materials for

Spatiotemporal dynamics of the proton motive force on single bacterial cells

Anaïs Biquet-Bisquert *et al.*

Corresponding author: Ashley L. Nord, ashley.nord@cbs.cnrs.fr; Francesco Pedaci, francesco.pedaci@cbs.cnrs.fr

Sci. Adv. **10**, eadl5849 (2024)
DOI: 10.1126/sciadv.adl5849

This PDF file includes:

Sections S1 to S10
Figs. S1 to S8

1 Microscope setup

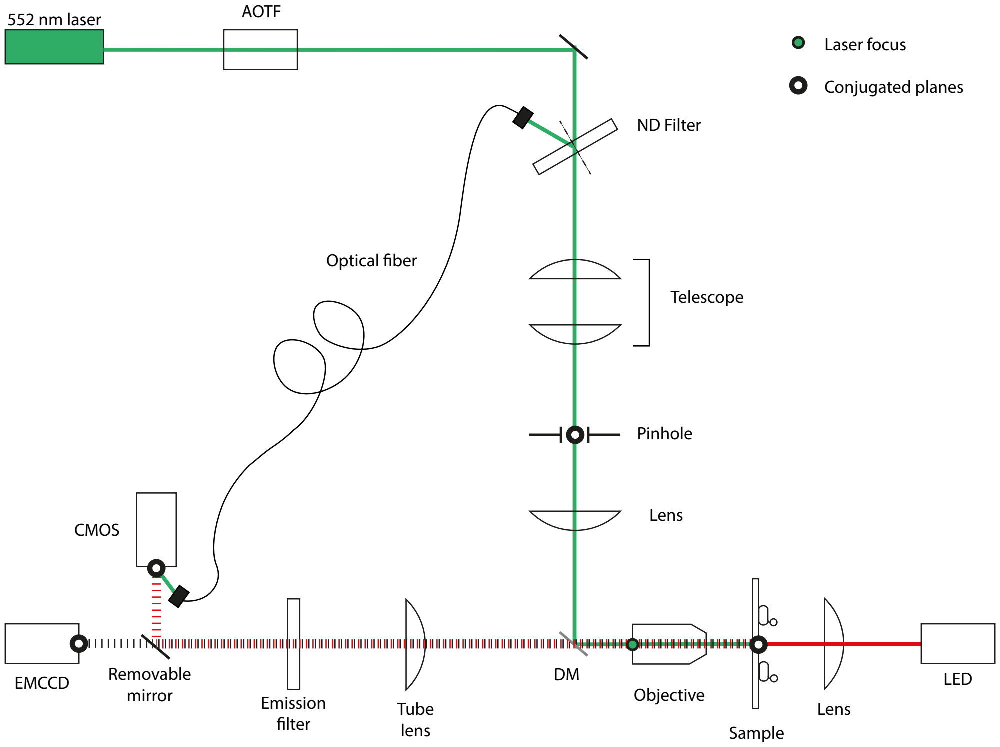

Figure S1: Schematic of the optical setup consisting of two illumination paths: brightfield and epifluorescence. In the first path (red) the sample was illuminated with a 660 nm LED and imaged with a 100× (1.45 NA) oil-immersion objective (Nikon) onto a fast CMOS camera (Optronics CL600x2/M). In the epifluorescent path (green), a 552 nm laser controlled by an Acousto-Optic Tunable Filter (AOTFnC-400.650-TN, AA Opto-electronic) is used to excite PR. The beam intensity was reduced via an ND filter and its diameter was controlled by lenses and a pinhole. The auto-fluorescence of the cells was imaged via a removable mirror onto a cooled Electron Multiplying Charge Coupled Device (EMCCD, iXon Ultra 897, Andor). Using an optical fiber collecting the reflected light from the ND filter, the laser intensity was imaged onto a corner of the CMOS, providing the state of the laser synchronously with the acquisition of the rotating bead.

2 Laser spot size measurement

SI Fig. S2a shows the position of the laser next to a cell at a distance of 4 µm, measured from the center of the laser spot to a rotating motor represented in red. At this distance, we observed no effect of the laser on the motor speed (SI Fig. S2c, red). On the contrary, the speed of the same motor, indicated in blue in SI Fig. S2b-c, follows the switching on and off of the laser when the center of the laser is placed on the cell, again at a distance of 4 µm from the motor. This indicates that the effective laser spot diameter is $\sim 8\,\mu m$. The laser spot size was also measured using a thin layer of fluorescent dye solution in water. The sample was prepared by placing a drop of $0.3\,g\,L^{-1}$ Rose Bengal (Sigma-Aldrich, 198250-5G) dye solution, previously sonicated for 30 min, between two cleaned coverslips and letting the solution sit for 10 min. The sample was illuminated with the laser (at a power of 6 mW mm^{-2}, the intensity used in the PMF spatial dynamics measurements) and imaged onto the EMCCD camera (SI Fig. S2d,e,f).

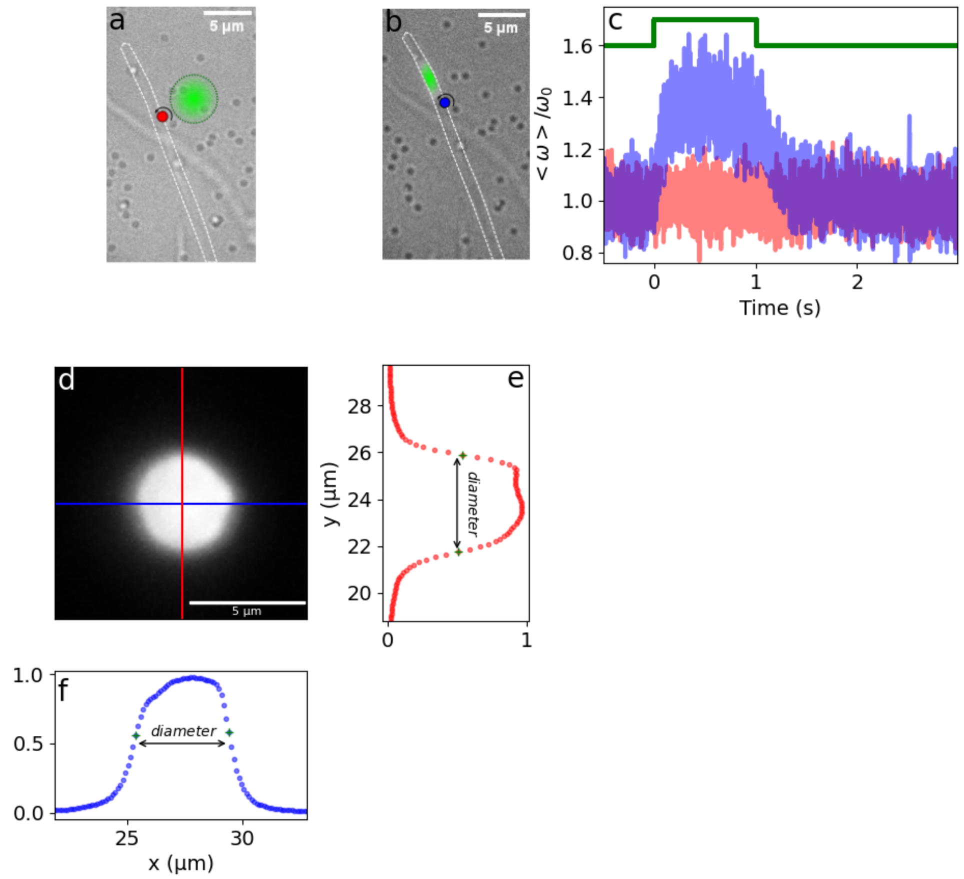

Figure S2: Measurement of the effective laser spot size. (a) Image of a filamentous *E. coli* cell (outlined in white) with a functioning motor labeled by a bead (in red). The laser (in green) is placed on the coverslip at a distance of 4 µm from the motor. (b) The laser spot is moved on the cell, 4 µm from the bead. (c) Normalized average speed response for 58 transitions of the motor from configuration (a) in red and configuration (b) in blue. The speed response was low-pass filtered by the Savitzky–Golay algorithm (5th order, $\sim$ 6 ms window). (d) Image of a thin layer of fluorescent Rose Bengal solution illuminated with the laser using a CCD camera. (e-f) Corresponding vertical and horizontal intensity profiles.

3 Error estimation

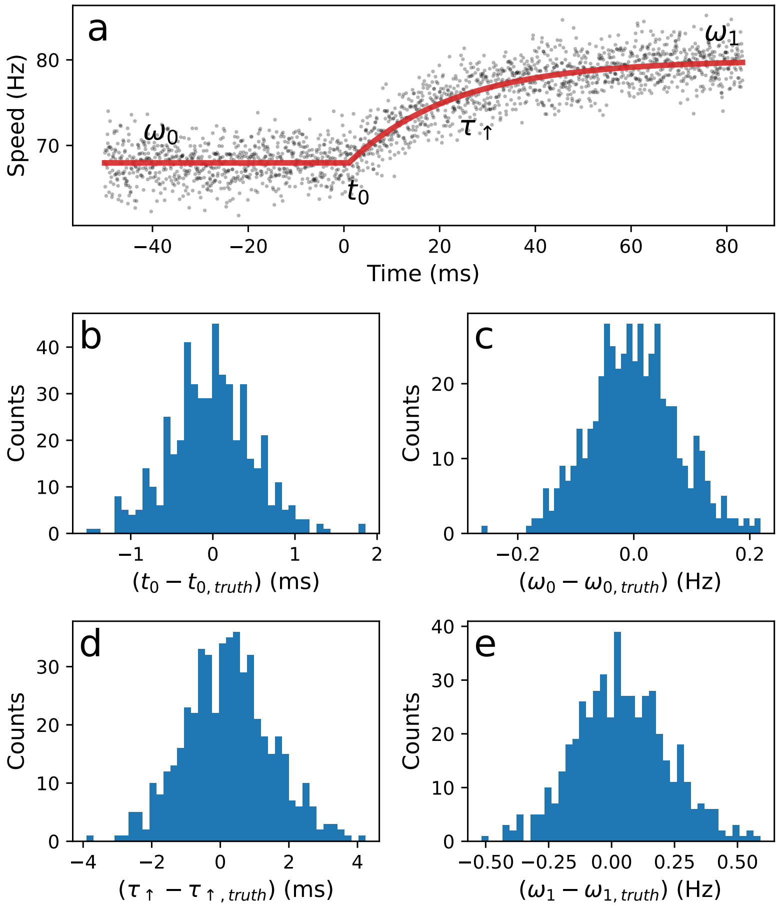

Figure S3: Resolution estimation.(a) A synthetic trace (dark points) is created mimicking the experimental charging response (averaged over laser transitions) at the laser-on edge, using a constant value followed by an exponential charging function (red line, see Eq.6 of the main text), to which a gaussian noise is added, with standard deviation equal to the one of the experimental trace. The fitting algorithm is challenged against 500 different instances of the noise. The distance distributions between the fitted parameters $(t_0, \omega_0, \tau_\uparrow, \omega_1)$ and the corresponding true ones, are indicated in panels (b),(c),(d), and (e), respectively.

4 Global statistics

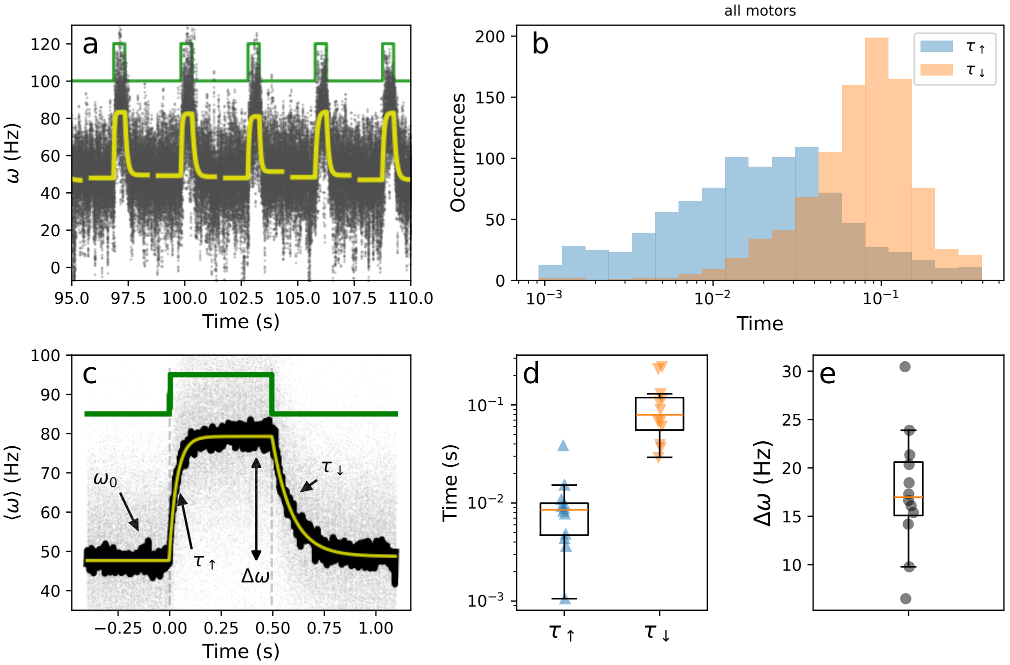

Figure S4: Global statistics. (a) BFM speed response of an *E. coli* cell expressing PR (grey) induced by a train of on-off rectangular laser pulses (green). A piecewise function composed of single exponential functions was fit to each speed transition generated by a laser pulse (yellow). Same as in Fig. 1e of the main text. (b) Histogram of the characteristic times, $\tau_{\uparrow}$ (green) and $\tau_{\downarrow}$ (red), extracted from 12 different motors and a total of 936 speed transitions. (c) Overlay of hundreds of laser-synchronised transitions (grey), mean of the transitions (black), and fit of the mean (yellow). Same as in Fig. 1e of the main text. (d) characteristic times $\tau_{\uparrow}$ (green) and $\tau_{\downarrow}$ (red) extracted from the average transition fit of 12 motors. (e) Increase in speed extracted from the average transition fit of 12 motors

5 Local PMF perturbation as a function of laser intensity

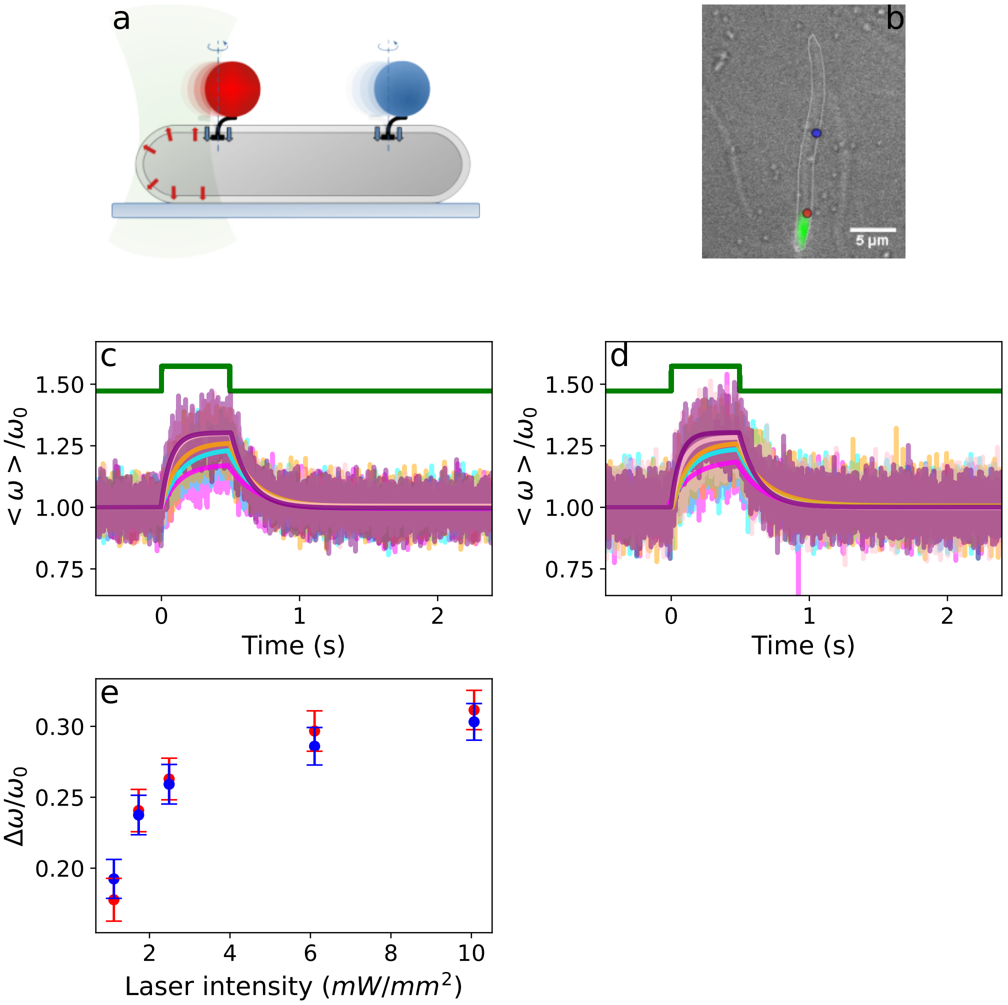

Figure S5: Two motors on the same cell react similarly to increasing PR excitation localized on one cell pole. (a) Schematic of a cell with two beads attached to the flagellar filaments and a laser illumination located at one pole. (b) Microscope image of the filamentous cell (outlined in white), the two motors (labeled by beads), and laser spot (green) (c,d) Normalized average speed traces of the proximal motor labeled in red (c), and of the distal motor labeled in blue (d), for different laser intensities (indicated by different colors): 1.1, 1.7, 2.5, 6.1 and 10.1 mW/mm^2. For each laser intensity, the average was obtained from 117, 109, 102, 119, and 134 transitions, respectively (Savitzky-Golay filter, 41 points). Lines are the fit to the data. (e) Relative increase of motor speed $\Delta\omega/\omega_0$ as a function of the laser intensity for the two motors (blue and red indicate the corresponding motors). At every laser power the two motors respond identically within noise.

6 Estimates of cellular electrical parameters from the circuit analysis

Walter et al [33]estimate V_r from the energy of NADH oxidation under physiological conditions ($\Delta G = 212$ kJ/mol, with a stoichiometry of $6H^+$ per electron pair) as $V_r \simeq 360$ mV. We further set $R_r = \beta R_s$, and use the circuit expressions $V_0 = R_\downarrow \frac{V_r}{R_r}$ and $R_\downarrow = (\frac{1}{R_r} + \frac{1}{R_s})^{-1}$ to obtain

$$V_r = V_0(1+\beta). \tag{S1}$$

In the following, we assume i) a linear relationship between PMF and BFM speed (with zero y-intercept), and ii) that the magnitude of the PMF of energized cells is ~ 150 mV [31]prior to oxygen reduction and with a BFM rotating at ~ 200 Hz. Therefore, the experimentally measured steady state speed after one to two hours in a sealed flow cell, $\omega_0 \simeq 50$ Hz, and the speed under PR excitation $\omega_1 \simeq 80$ Hz (Fig. 1 of the main text), correspond to a voltage $V_0 = 37.5$ mV and $V_1 = 60$ mV, respectively. From Eq. S1, we then obtain $\beta \sim 9$ in our experimental conditions. From $\tau_\downarrow = R_\downarrow C$ we obtain

$$R_s = \frac{\tau_\downarrow(1+\beta)}{\beta C} \tag{S2}$$

Using the measured value of $\tau_\downarrow \simeq 100$ ms, and the published value of $C \simeq 10^{-14} F$ [47,33],we get $R_s \simeq 1 \times 10^{13}\,\Omega$, and $R_r \simeq 9 \times 10^{13}\,\Omega$ (we note that the assumptions made in [33]led to $R_s \simeq R_r \simeq 1 \times 10^{14}\,\Omega$ to $1 \times 10^{15}\,\Omega$). Using the circuit expressions $V_1 = R_\uparrow(\frac{V_r}{R_r} + \frac{V_{pr}}{R_{pr}})$ and $R_\uparrow = (\frac{1}{R_r} + \frac{1}{R_s} + \frac{1}{R_{pr}})^{-1}$, we obtain the expression for V_{pr} as

$$V_{pr} = \frac{V_1(1+\beta)R_{pr} + V_1\beta R_s - V_r R_{pr}}{\beta R_s} \tag{S3}$$

Using $R_{pr} \simeq 1 \times 10^{12}\,\Omega$ (obtained from the expression $1/\tau_\uparrow - 1/\tau_\downarrow = 1/(R_{pr}C)$, see main text), we finally obtain $V_{pr} \simeq 62$ mV.

7 Analytical solution of the cable equation

The 1D cable equation for the quantity $A(x,t)$ (which can be voltage or concentration) can be written as function of space, x, and time, t, normalized to the characteristic length, λ, and characteristic time, τ, as,

$$\frac{\partial A}{\partial t} = \frac{\partial^2 A}{\partial x^2} - A + F_A(x,t). \tag{S4}$$

When the external source F_A is applied at a point x_o along a cable of length L, during the time window $(t_1 = 0, t_2)$, one can write $F_A(x,t) = \delta(x - x_o)[H(t) - H(t - t_2)]$, with $H(t)$ the unit step function. The analytical solution (shown in Fig. 4 of the main text) can be written as [39]

$$A(x,t)\,L = 1 - e^{-t} + 2\sum_{n=1}^{\infty} \frac{\phi_n(x)\phi_n(x_o)}{\mu_n}(1 - e^{-\mu_n t}) \qquad t \in [0, t_2] \tag{S5}$$

$$A(x,t)\,L = e^{-t}(e^{t_2} - 1) + 2\sum_{n=1}^{\infty} \frac{\phi_n(x)\phi_n(x_o)}{\mu_n}(e^{-\mu_n(t-t_2)} - e^{-\mu_n t}) \qquad t \in (t_2, \infty) \tag{S6}$$

with $\phi_n(x) = \cos(n\pi x/L)$ and $\mu_n = 1 + n^2\pi^2/L^2$, $(n = 1, 2, ..)$. Although the above solution was found in the context of cable theory [39],it can be applied to the diffusion problem, upon substitution of the relevant parameters.

8 Circuit and diffusion simulations

Here we show the results of the simulation of i) a simple circuit which models the spatial extension of the cell, and ii) of the diffusion equation with losses. This was done to include processes that are not present in the cable and diffusion equation (main text eq.3): a) the choice between a source of current and a source of voltage with internal resistance (SI fig.6 a,b,c), where eq.3 assumes a current source; b) the choice of using $k_{pr} = c.st$ or a saturable $k_{pr}(\rho)$ function of local concentration ρ, where eq.3 assumes the former. Here we show that the observed asymmetry in characteristic times ($\tau_\uparrow < \tau_\downarrow$) depends on these choices.

i) The circuit (SI Fig. S6a) is composed by two parallel RC sub-circuits, one of which includes the light-driven source. We consider both a voltage source V_{pr} with internal resistance R_{pr}, and a current source I_{pr}. The two sub-circuits correspond to two spatially separated parts of the cell (where two sensors can be located), connected by the cytoplasmic resistance R_i (internal). R_s is the membrane resistance (sinks), C_0 and C_1 (of the same value) indicate the membrane capacitance at the two locations where the voltage measurement is performed. To compare the results obtained with the two sources (voltage and current), we fix $I_{pr} = V_{pr}/(R_{pr} + R_{eq})$, where $R_{eq} = 1/(1/R_s + 1/(R_s + R_i))$ is the equivalent resistance seen by the source. In SI Fig. S6b, the values of the electrical components are the ones obtained from the analysis described in the main text (see figure legend). We assume a cylindrical cell of 0.5 μm radius, and a distance between the two sections of the cell, modeled by the two sub-circuits, of 20 μm in line with our measurements. In particular, this results in a cytoplasmic resistance $R_i = 10^7\,\Omega$, a value orders of magnitude smaller than the other resistances. With such small R_i, SI Fig. S6b shows that the two sub-circuits respond identically, as the voltage across C_0 and C_1 (continuous and dashed lines of both colors, respectively) perfectly overlap. Using a voltage source (blue continuous and dashed lines) introduces an asymmetry in the characteristic times ($\tau_\uparrow < \tau_\downarrow$, defined in the main text), which is not present with the current source (green continuous and dashed lines). To observe a difference between the transmembrane voltage measured across C_0 and C_1, we have to artificially increase R_i by several orders of magnitude. This results in the traces shown in SI Fig. S6c, where the voltage across C_1 (green and blue dashed lines, for both source types) reaches a lower plateau than the one across C_0.

ii) In SI Fig. S6d,e, we numerically integrate the 2D diffusion equation for the concentration $\rho(x,t)$ with losses k_s and sources k_{pr}

$$\frac{\partial \rho}{\partial t} = D_H \frac{\partial^2 \rho}{\partial^2 x} - k_s \rho + k_{pr} \tag{S7}$$

where $D_H = 9 \cdot 10^{-9}\, m^2/s$ is the Grotthuss diffusion coefficient. The integration is performed on a cylinder (periodic boundary conditions on one direction and zero Neumann on the other) of 35 μm length and 0.5 μm radius (which effectively makes the system 1D), where the source k_{pr} is spatially localized on one pole on the first 6 μm of the cell, and is temporally switched on at $t_0 = 0$ and off at $t_1 = 0.3$ s. The concentration is monitored at two points on the membrane, indicated by circles labeled C_1, C_2 in SI Fig. S6d, the first at the location of the source, and the second at a distance of 20 μm, to simulate the two farthest BFMs found in our measurements. The green continuous and dashed lines in SI Fig. S6e indicate the evolution of the concentration at C_0 and C_1, respectively, for a source modeled by a constant rate ($k_{pr}(x,t) = const. > 0$, for x at the excited pole and $t \in [t_0, t_1]$, otherwise $k_{pr}(x,t) = 0$). We also introduce a Hill-Langmuir saturation mechanism in the source, to

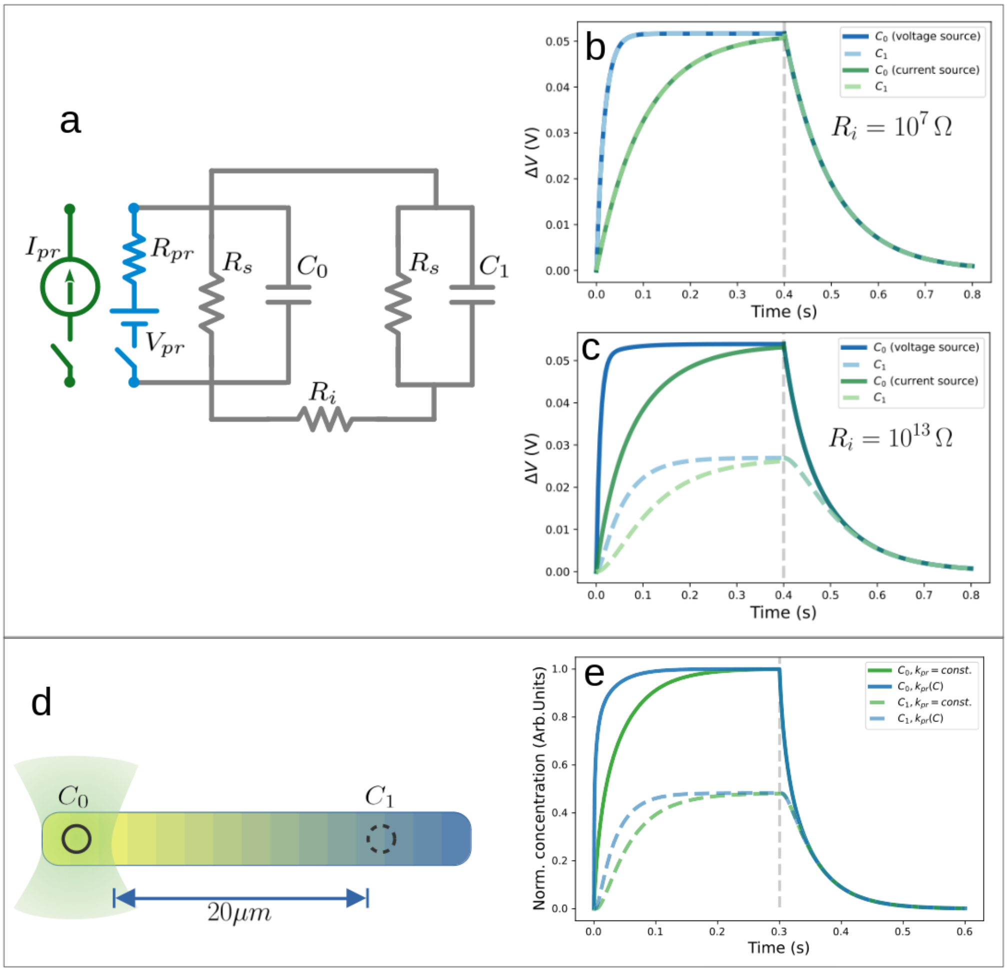

Figure S6: Circuit and diffusion simulations. (a) The circuit simulated is composed by two RC sub-circuits (membrane resistance from sinks R_s, and membrane capacitance C) connected by an internal cytosol resistance R_i (while the outer medium is considered conductive). We consider either a voltage (V_{pr}, R_{pr}, blue) or a current source (I_{pr}, green). (b) Circuit simulation with parameters obtained from the analysis described in the main text, where we fix $R_i = 10^7\,\Omega$ (the resistance of a cylindrical cell of radius $0.5\mu m$, length of 20 μm, and volume resistivity of 100 Ω cm [38, 47].The parameters used are $R_s = 10^{13}\,\Omega$, (so $R_i \ll R_s$), $C_0 = C_1 = 10^{-14}$ F. The blue lines (continuous and dashed) correspond to the voltage source with $V_{pr} = 0.062$ V, and $R_{pr} = 10^{12}\,\Omega$. The green lines (continuous and dashed) correspond to the current source, with $I_{pr} = V_{pr}/(R_{pr} + R_{eq})$, where $R_{eq} = 1/(1/R_s + 1/(R_s + R_i))$ is the equivalent resistance of the gray circuit in a). The continuous lines (blue and green) correspond to the voltage across C_0. The dashed lines (blue and green) correspond to the voltage across C_1. The voltage source introduces an asymmetry in the characteristic times ($\tau_\uparrow < \tau_\downarrow$), which is absent with the current source ($\tau_\uparrow = \tau_\downarrow$). (c) Same as (b), but with an increased internal resistance ($R_i = 10^{13}\,\Omega$, so $R_i = R_s$), which produces a reduction of the value of the plateaus reached by the voltage across C_1 (dashed lines), for both source types. (d) Diffusion simulations. Eq. S7 is integrated on a cylinder with diffusion coefficient $D_H = 9 \cdot 10^{-9}\,\mathrm{m}^2/\mathrm{s}$ and $k_s = 20\,\mathrm{s}^{-1}$. The source is located at one pole of the cell, and it is switched on for 0.3 s. The concentration is monitored at the two points C_0, C_1. (e) Evolution of the normalized concentration. Green lines correspond to the choice $k_{pr} = const. = 1$ (see text), while the blue lines correspond to the choice $k_{pr} = k_{pr}(\rho)$ (Eq. S8, with k'_{pr}=1 s^{-1}, $K = 10^{-4}$ and $h = 2$). Continuous and dashed lines indicate the measurement at point C_0 and C_1, respectively. An asymmetry in the characteristic times ($\tau_\uparrow < \tau_\downarrow$) appears with the choice $k_{pr} = k_{pr}(\rho)$ (blue lines). A clear difference is visible between the plateaus reached by ρ at C_0 and C_1. A difference in the plateau reached at C_1 with respect to C_0 is present, as for the electric circuit with low internal resistance in (c).

make k_{pr} a decreasing function of concentration ρ, using

$$k_{pr}(\rho) = k'_{pr}\left(1 - \frac{\rho^h}{K^h + \rho^h}\right) \tag{S8}$$

where k'_{pr} is the source rate at low concentration, K the equivalent of a dissociation constant, and h the Hill coefficient. In SI Fig. S6e, the blue continuous and dashed lines correspond to such choice of $k_{pr}(\rho)$. As in the case of the voltage source in the circuit above, this mechanism creates an asymmetry in the characteristic times ($\tau_\uparrow < \tau_\downarrow$) which is absent in the case of constant source rate.

Among the scenarios discussed above, our measurements of the spatial dynamics of the PMF are only compatible with SI Fig. S6b with a voltage source, where the voltage measured at the two distant sensors evolves identically, with asymmetric characteristic times. This is achieved by the voltage in the presence of a low cytoplasmic resistance R_i (with respect to the other resistances of the circuit) in the circuit model, and cannot be reproduced either by a circuit with R_i of the same order of the other resistances, or by diffusion of concentration ρ. In the case of diffusion, even considering the highest diffusion coefficient and reduced dimensionality (2D or 1D), the concentration develops a spatial gradient along the cell which produces, once measured by two sensors placed at a distance of 20 μm, traces with distinctly different plateaus, in contrast with our observations.

9 Proteorhodopsin expression

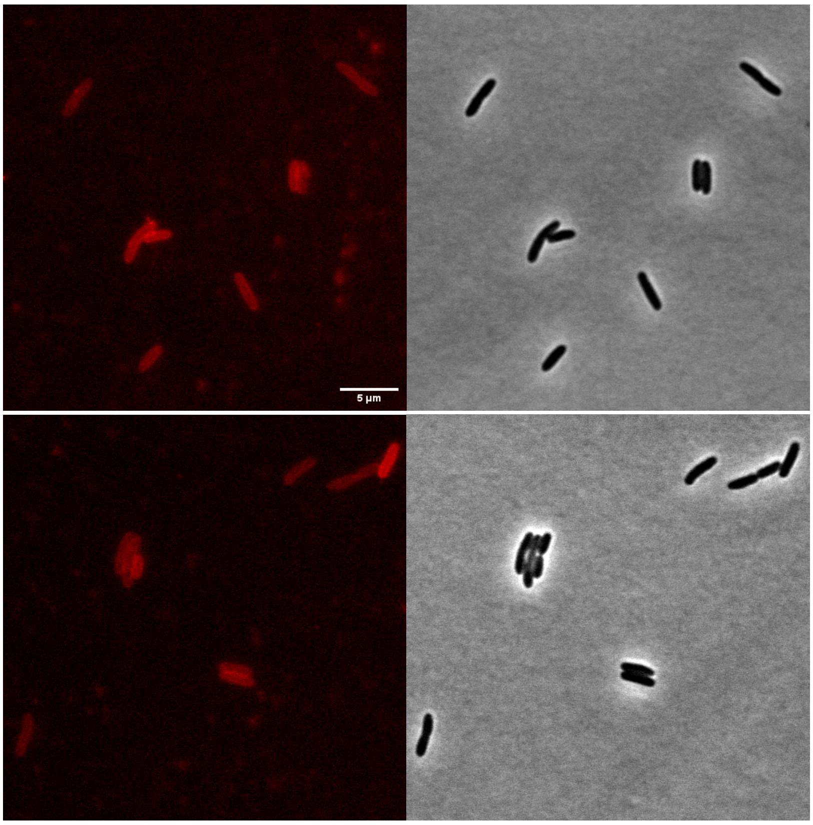

Figure S7: Proteorhodopsin expression. Left panels: bright field fluorescence images of *E.coli* cells expressing PR. The images were taken with 1.5 s, using a TexasRed emission filter. The cells were placed between the glass slide and an agar pad. The fluorescence signal from PR is weak, homogeneous, and more intense at the cell periphery, in agreement with the membrane localization of PR. Right panels: corresponding phase contrast images, taken with 2.5 s exposure time.

10 Lowering the efficiency of respiration

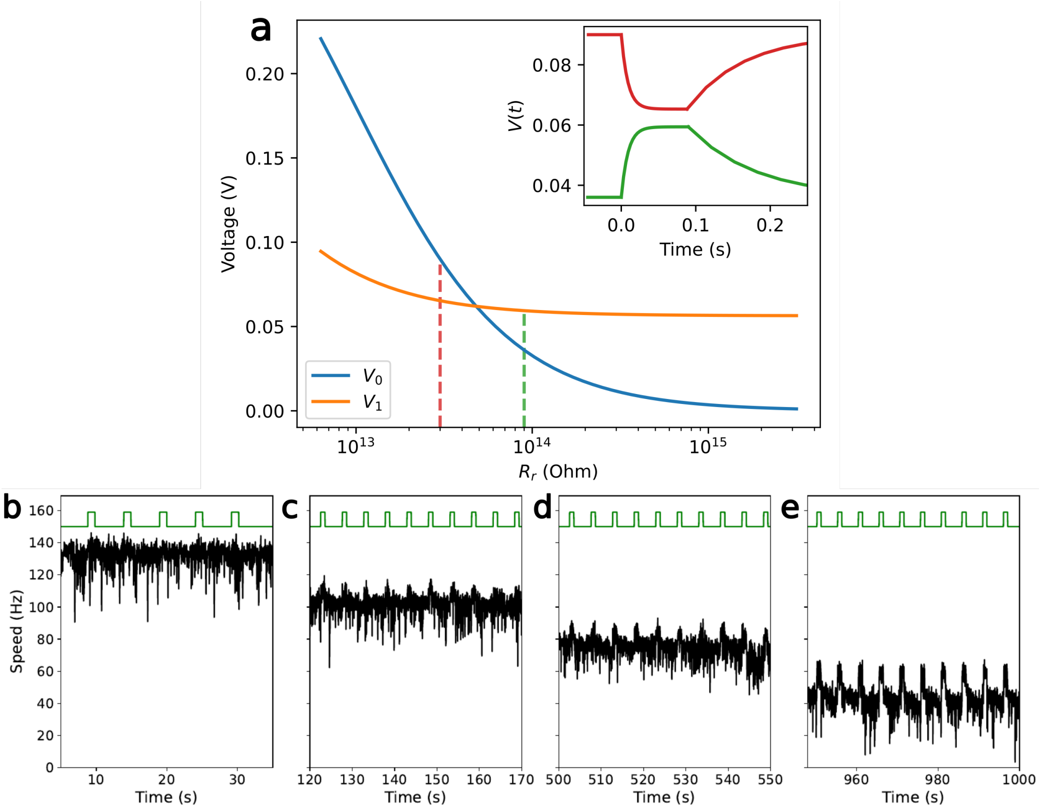

Figure S8: Respiration efficiency. a) Predictions of the circuit model (main text Eq.1,2) for the voltage levels V_0 (blue) and V_1 (orange) as a function of the respiration resistance R_r. The parameters used are those found in the main text: $R_s = 10^{13}\ \Omega$, $R_{pr} = 10^{12}\ \Omega$, $C = 10^{-14}\ F$, $V_{pr} = 0.062\ V$, $V_r = 0.360\ V$. The green and red dashed lines correspond to the values $R_r = 9 \cdot 10^{13}\ \Omega$ (the value found from the data) and $R_r = 3 \cdot 10^{13}\ \Omega$, respectively. The time trace of the corresponding solutions is shown in the inset, with the same color code. This shows that the response of the system to PR excitation, measured as $V_1 - V0$, changes direction beyond the critical value of R_r where $V_0 = V_1$. b-e) The measured effect of different concentrations of NaN_3 (from b to e: 0, 10, 15, 20 mM) over the speed of the motor shows qualitative agreement with the model, indicating that an increase in NaN_3 is analogous to an increase in R_r beyond its critical value. The decreasing value of V_1 in the experiment, marking a difference from the model, could be due to a change in stator number, which could not be kept fixed in these measurements.
